# A versatile, high through-put, bead-based phagocytosis assay for *Plasmodium falciparum*

**DOI:** 10.1038/s41598-017-13900-4

**Published:** 2017-10-31

**Authors:** Yukie M. Lloyd, Elise P. Ngati, Ali Salanti, Rose G. F. Leke, Diane W. Taylor

**Affiliations:** 1 Department of Tropical Medicine, Medical Microbiology and Pharmacology, John A. Burns School of Medicine, University of Hawaii at Manoa, Hawaii, USA; 2 Centre for Medical Parasitology, Department of Immunology and Microbiology, University of Copenhagen, Copenhagen, Denmark; 3 Department of Infectious Diseases, Copenhagen University Hospital, Copenhagen, Denmark; 4 Faculty of Medicine and Biomedical Sciences, Biotechnology Centre, University of Yaoundé I, Yaoundé, Cameroon

## Abstract

Antibody-mediated phagocytosis is an important immune effector mechanism against *Plasmodium falciparum*-infected erythrocytes (IE); however, current phagocytosis assays use IE collected from infected individuals or from *in vitro* cultures of *P. falciparum*, making them prone to high variation. A simple, high-throughput flow cytometric assay was developed that uses THP-1 cells and fluorescent beads covalently-coupled with the malarial antigen VAR2CSA. The assay is highly repeatable, provides both the overall percent phagocytosis and semi-quantitates the number of antigen-coupled beads internalized.

## Introduction

Opsonic phagocytosis is an important host immune effector mechanism against blood- stage *Plasmodium falciparum* ^1–4^. The intracellular parasite produces and displays a variety of variant surface antigens (VSA) on the surface of the infected erythrocytes and antibodies (Ab) against them help protect individuals from severe and symptomatic malaria^5–7^. Current assays for measuring Ab-mediated phagocytosis have used *P. falciparum*-infected erythrocytes (IE) collected from infected individuals or from *in vitro* cultures of *P. falciparum* that express a variety of VSA. Because of this variation, it is difficult to determine which VSA are important in phagocytosis, to repeat experiments, and to interpret results. Data support that members of the *P. falciparum* erythrocyte membrane protein 1 (PfEMP1) family are the key targets of naturally-acquired Ab and Ab-mediated phagocytosis of IE^1^. Thus, we sought to adapt a previously reported high-throughput phagocytosis assay that has proven useful in studies of bacteria^8^ and viruses^9^, to measure PfEMP1 Ab-mediated phagocytosis using the PfEMP1 antigen VAR2CSA to establish proof of concept. VAR2CSA causes IE to cytoadhere to chondroitin sulfate A on placental cells, thus only pregnant women, or women who have been pregnant, have high level of Ab to VAR2CSA^10–12^.

We sought to develop an assay that was high-throughput, highly versatile, reproducible, and could be used in any basic laboratory equipped with a simple flow cytometer. The steps in the assay include: 1) covalent coupling of recombinant *P. falciparum* proteins to fluorescent, carboxylated beads, 2) incubating the beads with plasma, 3) combining the opsonized beads with THP-1 monocyte cells, and 4) determining the percentage of monocytes that have internalized the beads by flow cytometry. Results showed that the assay meets our requirements and not only provides data on percent phagocytosis, but also gives semi-quantitative data on the number of antigen-coated beads internalized.

## Results and Discussion

During assay development, SPHERO^TM^ Carboxyl Fluorescent Particles, Yellow, Low-Intensity (CFL-5052-2, Spherotech, Lake Forest, IL) with a 5.0–5.9 μm diameter were selected, as they give a recordable fluorescent signal and are approximately the size of human erythrocytes. The beads were covalently coupled to full-length recombinant VAR2CSA using carbodiimide derivatives, EDC and Sulfo-NHS, and a protocol employed in multiplex bead-based LUMINEX assays^13^. Archival, de-identified plasma samples were used as the source of Ab. The samples were collected at delivery from Cameroonian women and pre-screened to determine the level of IgG to VAR2CSA. Plasma samples with high, intermediate, and low levels of IgG to VAR2CSA were tested. The assay was optimized using undifferentiated THP-1 cells, that only express CD64 (FcγR I), CD32 (FcγR II), and not CD16 (FcγR III) and CD36^2^. THP-1 monocytes were maintained below 5 × 10^5^ cells per ml of culture to ensure consistent surface receptor expression. Prior to use, THP-1 cells were immuno-stained for the above receptors to confirm their surface phenotype (Supplementary Fig. S1). The viability of THP-1 cells was >98% at the beginning of the assay (Bio Rad TC20 cell counter) and >98% at the time of data acquisition (7-AAD viability dye, eBioscience).

**Figure 1:**
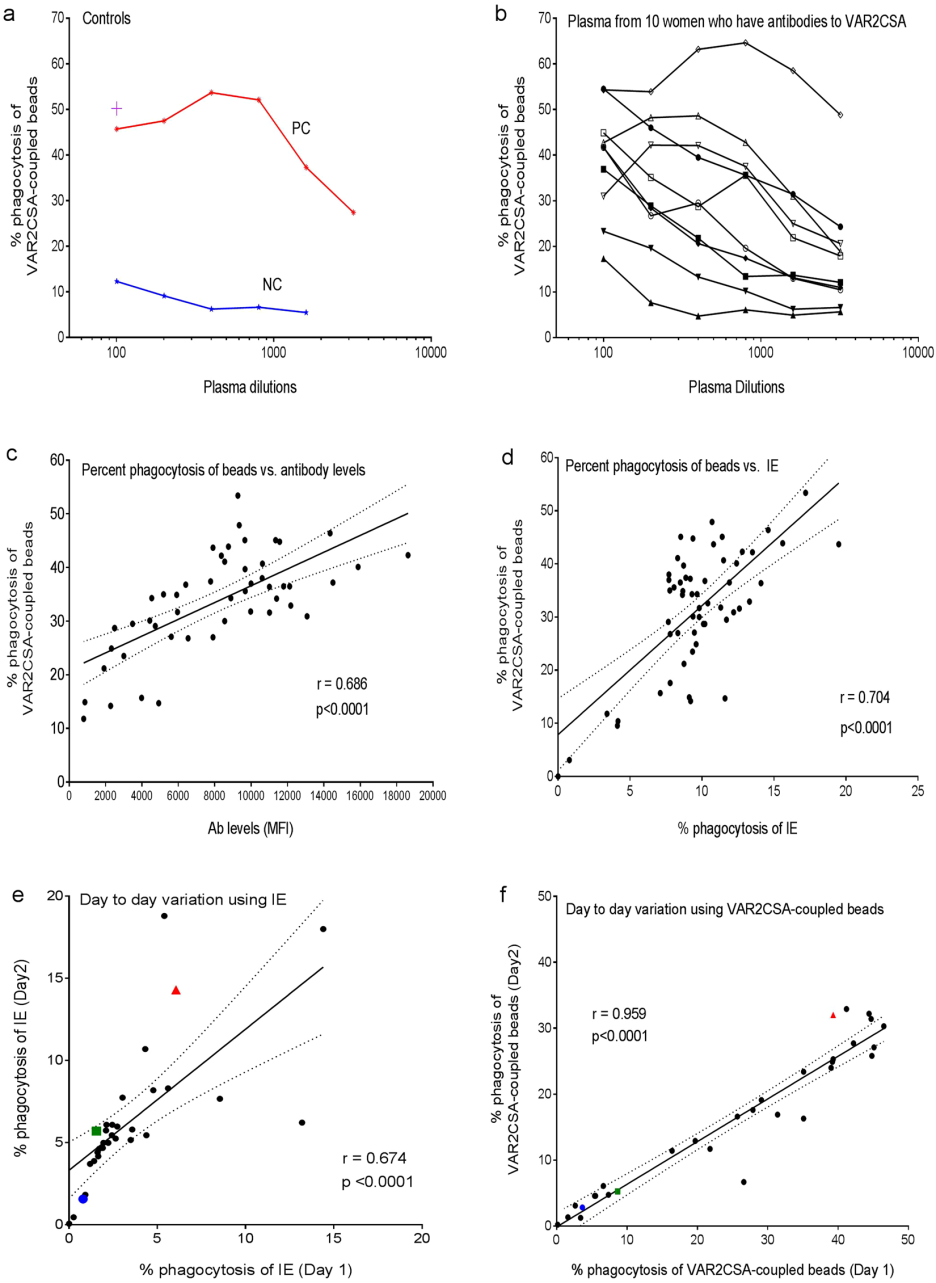
Detection of FcγR-mediated phagocytosis of VAR2CSA-coupled fluorescent beads. (**a**) The percent of THP-1 cells that internalized beads treated with serial dilution of the positive control (PC) (red line) consisting of pooled plasma from multigravida women known to have high IgG levels to VAR2CSA and the negative control (NG) (blue line) consisting of pooled plasma from nulligravidae who have Ab to multiple PfEMP1 antigens, but not to VAR2CSA. The purple cross (+) represents percent phagocytosis of beads coupled with human IgG. (**b**) Percent phagocytosis of VAR2CSA-coupled beads incubated with serial dilution of plasma from 10 women with different levels of Ab to VAR2CSA. Figure 1a and b show representative results from replicate experiments. (**c**) Correlation between the percentage of THP-1 cells that internalized beads coupled with VAR2CSA (Y-axis) versus corresponding anti-VAR2CSA antibody levels measured in median fluorescent Intensity (MFI) units (X-axis) (n = 57). (**d**) Comparison of percent phagocytosis of VAR2CSA-coupled beads (Y-axis) and IE (X-axis) (n = 50). (**e**) Comparison of day-to-day variation of phagocytosis of IE (n = 31). (**f**) Comparison of day-to-day variation of phagocytosis of VAR2CSA-coupled beads (n = 31). Correlations were determined by Pearson’s correlation coefficient. P-values of < 0.05 were considered significant.

The first step in assay optimization was to determine the optimal dilution of plasma to use (Fig. 1a). Two positive controls were used, including beads coupled with purified human IgG and VAR2CSA-coupled beads incubated with pooled plasma from multigravida women with high IgG levels to VAR2CSA. VAR2CSA-coupled beads incubated with plasma from nulligravid women (NG), who have Ab to multiple *P. falciparum* PfEMP1/VSA, but not VAR2CSA, were used as the negative control. Percent phagocytosis was >50% for the PC and 10% for NG. The titration of plasma from 1:100 to 1:3,200 using PBS from 10 women showed that the percentage of THP-1 cells that internalized VAR2CSA-coupled beads declined with increasing plasma dilution (Fig. 1a); confirming a relationship between amount of IgG Ab to VAR2CSA in plasma and phagocytic activity. Using plasma dilutions of 1:10 and 1:50 did not increase phagocytosis. Although the highest percent phagocytosis was obtained at a 1:100 dilution, plasma dilutions that gave the best discrimination among the samples were 1:400 to 1:800. In a follow-up study using plasma from 50 Cameroonian multigravid women who had Ab to VAR2CA, a good correlation between their anti-VAR2CSA Ab levels and percent phagocytosis was found, r = 0.6855, p < .0001 (Fig. 1b). Additional studies confirmed that phagocytosis was mediated by Abs to VAR2CSA using several specificity controls, e.g., incubating THP-1 cells with VAR2CSA-coupled beads treated with plasma from Cameroonian male (who have Ab to multiple VSA, but not VAR2CSA) and US pregnant women, as well as, with beads coupled to an irrelevant antigen, keyhole limpet hemocyanin (KLH; G-Biosciences) (Supplementary Fig. S2). Finally, time-lapse microscopy verified that THP-1 cells selectively phagocytized opsonized VAR2CSA-coupled beads and not KLH-coupled beads (Supplementary Fig. S3). Similar results were obtained after reversing the coupling, showing that THP-1 cells did not favour one bead type (colour) over another.

Next, the new bead-based assay was compared with other standard assays used in the field. First, it was compared with a standard phagocytic assay that uses IE obtained from *in vitro* cultures^14^. In the assay, CS2 *P. falciparum* IE (expressing VAR2CSA) were obtained from *in vitro* cultures, enriched by 0.7% gelatin flotation, stained with the nucleic dye dihydroethidium (DHE) and the cytosolic dye Cell Trace (Invitrogen, Waltham, MA), and incubated with THP-1 cells prior to analysis by flow cytometry^15^. Results from the two assays were significantly correlated (r = 0.70, p < 0.0001), with a higher percent phagocytosis found for many samples in the bead-based assay (Fig. 1c). It is possible the density of VAR2CSA is higher on the surface of beads compared to IE, thereby, enhancing sensitivity. Next, day-to-day variation of the new and standard phagocytosis assays was compared (Fig. 1d,e). Higher variation was found in the assay using IE (r = 0.67, p < 0.0001) than with the bead-based method (r = 0.96, p < .0001). Finally, the bead-based phagocytic assays was compared with the traditional cell surface staining assay using CS2 trophozoite-enriched IE labeled with a nucleic acid stain and then incubated with plasma, followed by FITC-Goat-anti-human IgG^16^ (Supplementary Fig. S4). A strong correlation between the two assay results was observed (r = 0.69, p = 0.0003). Thus, the new assay compared well with, and was significantly easier and more reproducible than, assays that are currently being used.

**Figure 2:**
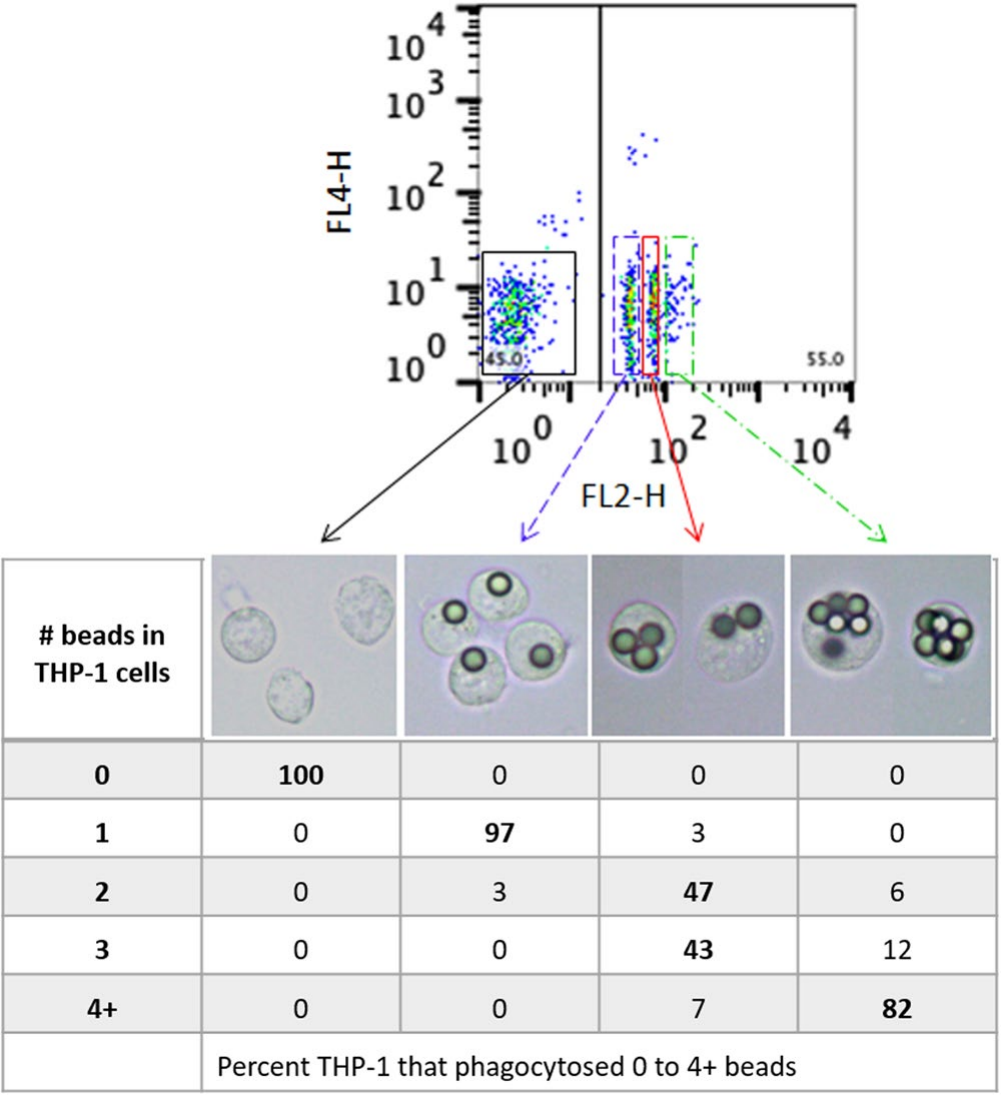
Sorting of the four major peaks by fluorescent intensity. THP-1 cells were incubated with VAR2CSA-coupled fluorescent beads opsonized with pooled plasma from multigravid women with high levels of Ab to VAR2CSA. THP-1 cells were gated on FSC and SSC and then fluorescence was detected in channels FL2 and FL4. Four fractions were seen: THP-1 cells that did not internalize beads (black box) and 3 peaks of THP-1 cells that had phagocytosed fluorescent beads (coloured boxes). The photomicrographs show representative THP-1 cells in each fraction. Table reports the percentage of THP-1 cells that had internalized different numbers of beads in the fractions after incubation with a pool of plasma from multigravid women. Data in the Table are based on sorted cells in wet-mount preparations visualize microscopy (n = 17 to 69 cells counted per fraction).

In the optimized assay, antigen-coupled fluorescent beads within the THP-1 cells were detected in the FL-2 channel paired with the FL-4 channel and data were recorded as median fluorescence intensity (MFI) (Fig. 2). Four distinctive cell populations were seen, namely, THP-1 that had not internalized beads and three populations that had (Fig. 2). The three major phagocytic populations were sorted using FACS Aria and results showed the THP-1 cells in the peaks had internalized one, 2–3, or more than 5 VAR2CSA-coupled beads, respectively. Thus, the assay not only identified the percentage of THP-1 cells that phagocytized opsonized VAR2CSA-coupled beads (requires a single laser, FL2), but by setting parameters on a second channel (FL4), also semi-quantitated the number of opsonized beads phagocytized.

In conclusion, the bead-based assay for measuring Fcγ-mediated phagocytosis has advantages over assays using intact IE, namely, fluorescent beads are inexpensive ($0.07 USD per sample), the coupled-beads require minimal antigen (200 ng/10^6^ beads), and the beads are stable for a long period of time; the assay requires neither *in vitro* cultured parasites that are expensive to grow nor purified IE that lead to inter-assay variation. The use of THP-1 cells maintained under standard conditions is a good source of well-characterized phagocytes. One disadvantage is that only one antigen, and not the whole plethora of VSA is studied, although several different PfEMP1 proteins can be coupled to the same bead type.

A strength of the assay is its versatility. By coupling different recombinant VSA proteins, the repertoire of naturally-acquired Ab can be determined, both for different antigens and different domains. The new assay has many additional uses, e.g., antigen-competition assays using beads in distinct fluorescent spectra coupled to different antigens; measuring Ab-mediated phagocytosis against liver stage (e.g., LSA1), merozoite (e.g., MSP1), and sexual stage (e.g., Pf25) antigens by simply coupling stage-specific antigens to beads; and studies of *P. vivax* that does not grow in continuous culture. Recently, Osier *et al*. reported that opsonic phagocytosis of *P. falciparum* merozoites correlated with protection in children and suggested that Ab to MSP2 and MSP3 were important. Instead of isolating merozoites, MSP2 and MSP3 could be coupled to beads and a high through-put study conducted to confirm and extend these interesting results. Thus, the new bead-based assay has the potential for becoming an important new tool in malaria research.

## Methods

### Ethical approval

This study was approved by the Institutional Review Board (IRB) at the University of Hawaii at Manoa (protocol CHS# 22325). The archival plasma samples used in the study were collected between 1996 and 2001 in a study approved by the Ethics Committee, Ministry of Health, Cameroon and the IRB, Georgetown University. At the time of sample collection, written informed consent was obtained from all participants. All experiments were performed in accordance with relevant guidelines and regulations.

### Study samples

The archival, de-identified plasma samples used were collected at delivery from Cameroonian women residing in Yaoundé, Cameroon. Details of the study have been described^17^. Plasma from sympatric males and nulligravid women were used as negative controls, as well as plasma samples from US pregnant women who had not be exposed to malaria (obtained from the University of Hawaii Biorepository). All plasma samples were heat-inactivated for 30 minutes at 56 °C before use to inhibit complement activity.

### Measuring antibody levels to VAR2CSA antigens

Plasma samples from pregnant women were screened for IgG to VAR2CSA using a multiplex anayte-platform (MAP) assay that has been described previously^13,18^. In brief, full-length recombinant VAR2CSA (FCR3 line) was covalently-coupled to MAGPLEX beads using saturating amounts of VAR2CSA. Then, 2000 antigen-coupled beads were incubated with 30 μl of plasma diluted 1:200 using PBS for 1 hour at room temperature. The beads were then washed twice with PBS with 0.05% Tween, once with PBS with 1% bovine serum albumin and incubated with 100 μl of 2 μg/ml phycoerythrin-conjugated goat anti-human IgG (Jackson ImmunoResearch) for 1 h. The beads were washed twice with PBS with 0.05% Tween, once with PBS with 1% bovine serum albumin, and the level of fluorescence was measured using a LiquiChip 200 analyzer (Luminex Corp.) programmed to analyze a minimum of 100 beads per spectral address, DD Gate 7500–15000. Results were expressed in MFI units.

### *In vitro* culturing of *P. falciparum* IE and isolation of trophozoites

The CS2 line of *P. falciparum*, that constituently expresses VAR2CSA, was cultured *in vitro* in human type O-positive erythrocytes at 5% haematocrit. Parasite cultures were maintained in a humidified incubator (2% O_2_, 8% CO_2_, balance N_2_) at 37 °C. Medium was replaced daily. The IE were synchronized every week using 40–80% Percoll (Sigma) density gradient centrifugation^19^. Cultures were routinely tested by PCR for mycoplasma using the e-Myco^TM^ Mycoplasma PCR Detection Kit. For the erythrocyte surface binding assay, trophozoite-stage IE were enriched to 80–90% using magnetic columns^16,20^. For the phagocytosis assays, trophozoite-stage IE were enriched using a 0.7% gelatin (Porcein Gelatin A, Sigma) cushion dissolved in Roswell Park Memorial Institute (RPMI) medium 1640 for 45 minutes at 37 °C^19^. Trophozoite-stage IE were isolated by collecting the “funnel cloud” above the gelatin gradient, providing 40–50% trophozoite-stage IE.

### IE Surface binding

Binding of IgGs to CS2-IE was measured by flow cytometry^16^. Enriched, trophozoite-stage IE were incubated with the nucleic stain Syto61 (150 µl per 3 million cells, diluted 1:2,500 with PBS, Invitrogen) for 15 minutes in the dark at room temperature. IE were washed three-times with PBS and then 50 µl containing 5 × 10^5^ IE was added to V-bottom microtiter wells. The plasma used in the study was heat inactivated and pre-adsorbed twice with normal erythrocytes before use. The IE were incubated with 5 µl of plasma, at a dilution of 1:10 with PBS for 30 minutes at room temperature. Following incubation, IE were washed three times with PBS with 1%BSA and then incubated with 100 µl of 1.5 mg/ml FITC-labeled, goat Fab’_2_ fragment anti-human IgG gamma specific Ab diluted at 1:800 (Invitrogen) for 30 minutes at 4 °C. Samples were washed three times with PBS, fixed with 200 µl of 2% paraformaldehyde in PBS and immediately analyzed by flow cytometry (FACS Calibur, BD Biosciences). Erythrocytes were initially gated using forward scatter (FSC) and side scatter (SSC) density plots (CELL Quest software, BD Biosciences). Then, IE were gated for the Syto61 fluorescence signal. A minimum of 10,000 IE were acquired. Surface staining was measured as the geometric median fluorescence intensity (gMFI) for the IE population and the gMFI of uninfected erythrocytes was subtracted. Data were analyzed using the FlowJo (ver 10.6.0) software. The 3D7 strain of *in vitro* cultured *P. falciparum* parasites was used as a specificity control to assess the binding of Ab to non-VAR2CSA proteins expressed on the surface of IE.

### THP-1 culture

THP-1 cells (ATCC), a human monocyte cell line, were maintained in RPMI 1640 medium (GIBCO) supplemented with 10% heat-inactivated Foetal Bovine Serum (FBS, HyClone), 1% penicillin-streptomycin (GIBCO), 1% L-Glutamine (GIBCO), and 0.05 mM 2-Mercaptho-Ethanol (2ME, Sigma) at 37 °C in a 5% CO_2_ incubator. Cell densities were kept below 5 × 10^5^ per ml to maintain consistent cell surface expression of Fc receptors (FcγRs). Prior to use, cell surface receptor expression of CD64/FcγRI (Anti-Human CD64 PE, Clone 10.1), CD32/ FcγRII (Anti-Human CD32 FITC, Clone 6C4), and CD16/FcγRIII (Anti-Human CD16 PE, Clone eBioC316) were determined by flow cytometry using FITC or PE-labeled anti-human Ab to these receptors (eBioscience). The day before each experiment, culture medium was changed. Only cultures with >98% viability were used in the phagocytosis assays. Cultures of THP-1 were also routinely tested for mycoplasma as described above.

### *In vitro* IE Phagocytosis Assay

Gelatin-enriched, trophozoite-stage IE (up to 20 × 10^6^ total) were incubated with 1 ml of 5 μg/ml of dihydroethidium (DHE; Molecular Probes, Life Technologies) and 1 μM of Cell Trace^TM^ Far Red (Molecular Probes, Life Technologies) for 20 minutes in the dark. Cells were washed twice with RPMI with 2% FBS, and 1 × 10^6^ of stained IE were aliquoted into 96-well V-bottom wells (Corning) and incubated with 30 μl of various dilutions of heat-inactivated plasma. The plates were incubated for 1 hour on a Barnstead Lab-line titer plate shaker (Model #4625, Melrose, IL) at speed 3. Subsequently, IEs were washed twice with 200 μl of RPMI with 2% FBS and incubated with 50 μl of 1 × 10^5^ THP-1 cells (IE-to-monocyte ratio of 10:1) for 45 minutes in a 37 °C incubator (5% CO_2_). Following incubation, the 96-well plates were immediately placed on ice and 200 μl of ice-chilled PBS was added to each well to stop phagocytosis. THP-1 cells were promptly analyzed by flow cytometry (FACS Calibur, BD Biosciences) and gated by size and granularity on FSC and SSC plots (CELL Quest software, BD Biosciences). Data for at least 3,000 THP-1 cells were recorded for each sample. Data were analyzed using the FlowJo (ver 10.6.0) software. The percent phagocytosis equals the proportion of THP-1 cells that were positive for DHE and Cell Trace staining. Additional controls for the IE phagocytosis assays included: 1) non-opsonized IE; and 2) opsonized IE incubated with THP-1 at 4 °C.

### Coupling of fluorescent beads to *P. falciparum* antigen

One million yellow, carboxylated, fluorescent beads (CFL-5052-2; 5.0-5.9 μm, Spherotech) were covalently coupled with 200 ng of VAR2CSA or keyhole limpet hemocyanin (KLH; 200ng/million beads) using the carbodiimide reaction^13^. Beads were resuspended by vortexing and sonicating for 20 seconds before transfer to a microfuge tube. Beads were then pelleted by micro-centrifugation at 18,000 × g for 2 minutes. Supernatant was removed, beads were resuspended in 100 µl of ddH2O by vortexing and sonication for 20 seconds. Beads were again pelleted at 18,000 × g for 2 minutes and the supernatant was replaced with 80 µl of activation buffer (0.1 M NaH_2_PO_4_, pH 6.2). Ten microliters of freshly-prepared Sulfo-N-hyroxysulfosuccinimide (Sulfo-NHS) and 10 µl of 1-ethyl 3-(3-dimethylaminopropyl) carbodiimide hydrochloride (EDC; Pierce) solutions (10 mg into 200 µl of ddH_2_O) were added to each tube, gently mixed by vortexing, and incubated for 20 minutes in the dark, with gentle mixing by vortexing every 10 minutes. Activated beads were pelleted, supernatant was removed and beads were resuspended in 100 µl of coupling buffer (0.05 M MES, pH 7.0) by vortexing and sonicating for 20 seconds. After two washes with the coupling buffer, beads were resuspended in 100 µl of coupling buffer, vortexed and sonicated, and an optimal amount of antigen was added. The coupling reaction was mixed by votexing only and incubated at room temperature for 4 hours or at 4 °C overnight on a plate shaker at speed 6. Following incubation, coupled beads were pelleted at 18,000 × g for 2 minutes and supernatant was replaced with 1 ml of blocking/storage buffer (50 ml PBS-1%BSA, 10 µl Tween 20, 25 mg or 125 µl of a 20% solution Sodium Azide). A total of two washes was performed. Coupled beads were finally resuspended in blocking/storage buffer at 25,000 beads/µl and stored in the dark at 4 °C until used.

### *In vitro* Bead Phagocytosis Assay

First, 100,000 VAR2CSA-coupled beads were incubated with diluted, heat-inactivated plasma in a sterile 96-well V-bottom plate (Corning) at a total volume of 30 μl. The plate was covered with foil to prevent fluorescent beads from photobleaching and the plate was placed on a plate shaker at speed 5 for 1 h. Following two washes with PBS, beads were incubated with RPMI 50 μl containing 2 × 10^4^ THP-1 cells (beads-to-monocyte ratio of 5:1) for 45 minutes in a 37 °C incubator (5% v/v CO_2_). Then, the 96-well plates were placed on ice and 200 μl of chilled PBS was added to each well to stop phagocytosis. Samples were immediately read on FACS Calibur (BD Biosciences). THP-1 cells were gated by size and granularity on FSC and SSC plots (CELL Quest software, BD Biosciences). Data for at least 3,000 THP-1 cells, or at least 15% of total THP-1 cells in the well, were recorded for each sample. Data were analyzed using the FlowJo (ver 10.6.0) software. Percent phagocytosis was the proportion of THP-1 cells that were positive for fluorescence in the FL-2 channel. THP-1 cells in three major fluorescence peaks were visualized by setting the FL-2 channel against the FL-4 channel. To determine the cellular contents of the three peaks, the peaks were sorted using a FACS Aria (BD Biosciences). Each of the peaks was examined using wet-mount microscopy and the number of THP-1 cells that had engulfed 0, 1, 2, 3, 4+ fluorescence beads were tallied by two researchers. Photomicrographs of representative fields were taken using a 3.3MPX Digital Microscope Camera (ImagingPlanet, Santa Barbara, CA).

### Time-lapse Fluorescence Microscopy

For the video of the time-lapse fluorescent microscopy of phagocytosis, 2 × 10^4^ or 1 × 10^5^ THP-1 cells were plated into flat-bottom, polylysine-coated, 96-well plates (LabTek, MatTek). Then, 100,000 or 500,000 yellow or nile blue (CFL-5065-2; 5.0-5.9μm, Spherotech) fluorescent beads coupled with VAR2CSA or KLH were added to the adherent THP-1 cells. Phagocytosis was immediately imaged using an automated IX-81 microscope (Olympus, Japan) with a WSKM stage top incubator (Tokai Hit, Japan) that kept the cells at 37 °C and 5% CO_2_. Cells were imaged at a magnification of 320X every 30 seconds for 1 hour. For each time point, three images were taken in sequence, one for bright field, one using a GFP filter set, and one using a TxRed filter set. All images were captured using an Orca-R2 C10600 camera (Hamamatsu, Japan) controlled by Metamorph v.7.8.1 software (Molecular Devices, Downington, PA) which combined the images collected every 30 seconds into the video.

### Statistical Data Analysis

Data analyses was performed using Microsoft Excel and Prism version 7.03 (Graph Pad Software Inc.). Correlations were examined by Pearson’s Correlation Coefficient. P-values of < 0.05 were considered statistically significant.

## Electronic supplementary material

Supplementary Information

Supplementary Information

Supplementary Information
