# Supplementary material for: A versatile, high through-put, bead-based phagocytosis assay for Plasmodium falciparum
Source: Sci Rep. 2017 Oct 31;7:14705. doi: 10.1038/s41598-017-13900-4 (PMC5665980; doi:10.1038/s41598-017-13900-4)
Supplement: Supplementary file 3 — Supplementary Information [file 41598_2017_13900_MOESM3_ESM.pdf]

Supplementary Figures

Running Title: Bead-based phagocytosis assay for malaria

Full Title: A versatile, high through-put, bead-based phagocytosis assay for *Plasmodium falciparum*

Yukie M. Lloyd[1], Elise P. Ngati[1], Ali Salanti[2], Rose G.F. Leke[3] and Diane W. Taylor[1]

Affiliation:

1. Department of Tropical Medicine, Medical Microbiology and Pharmacology, John A. Burns School of Medicine, University of Hawaii at Manoa, Hawaii, USA.
2. Centre for Medical Parasitology, Department of Immunology and Microbiology, University of Copenhagen, Denmark; Department of Infectious Diseases, Copenhagen University Hospital, Denmark.
3. Faculty of Medicine and Biomedical Sciences, Biotechnology Center, University of Yaoundé I, Yaoundé, Cameroon.

Supplemental Figure S1

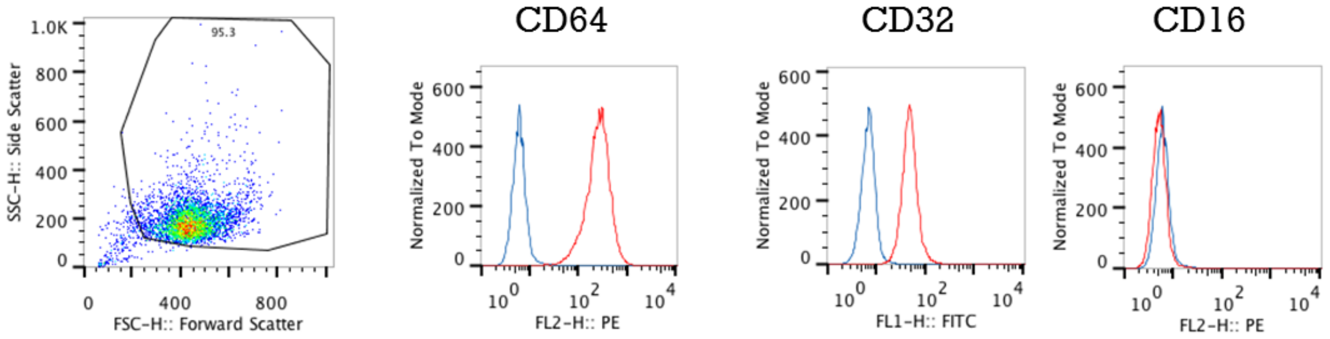

Supplemental Figure S1. The level of FcγR expression on THP-1 cells. THP-1 cells were first gated for FSC and SSC and then FcγRI (CD64), FcγRII (CD32), and lack of FcγRIII (CD16) expression was confirmed by flow cytometry (shown in red). Isotype controls are shown in blue.

Supplemental Figure S2

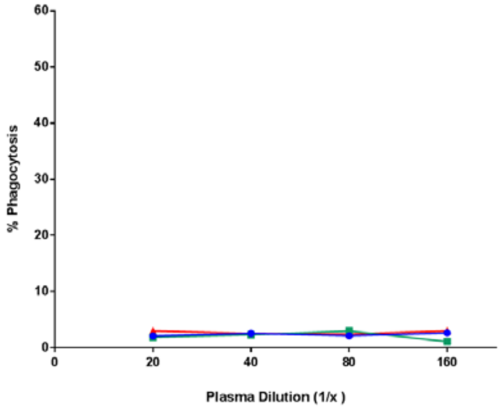

Supplemental Figure S2. Specificity Controls.
The percent of THP-1 cells that phagocytosed beads covalently coupled to keyhole limpet hemocyanin (KLH) that had been incubated with different dilutions of: 1) a pool of plasma from multigravidae with high levels of VAR2CSA IgG (red line), 2) a pool of plasma from nulligravidae (blue line), and 3) a pool of plasma from US pregnant women who had never been exposed to malaria (green line). These negative controls were part of the experiment shown in Fig. 1 A and B.

Supplemental Figure S3. Time-lapse video using fluorescence microscopy. (A) Video showing phagocytosis of VAR2CSA-coupled beads (red) and KLH-coupled beads (green) that had been incubated with positive control plasma. (B) Video showing the reverse coupling. This video shows the phagocytosis of VAR2CSA-coupled green beads and KLH-coupled red beads after incubation with the positive control plasma.

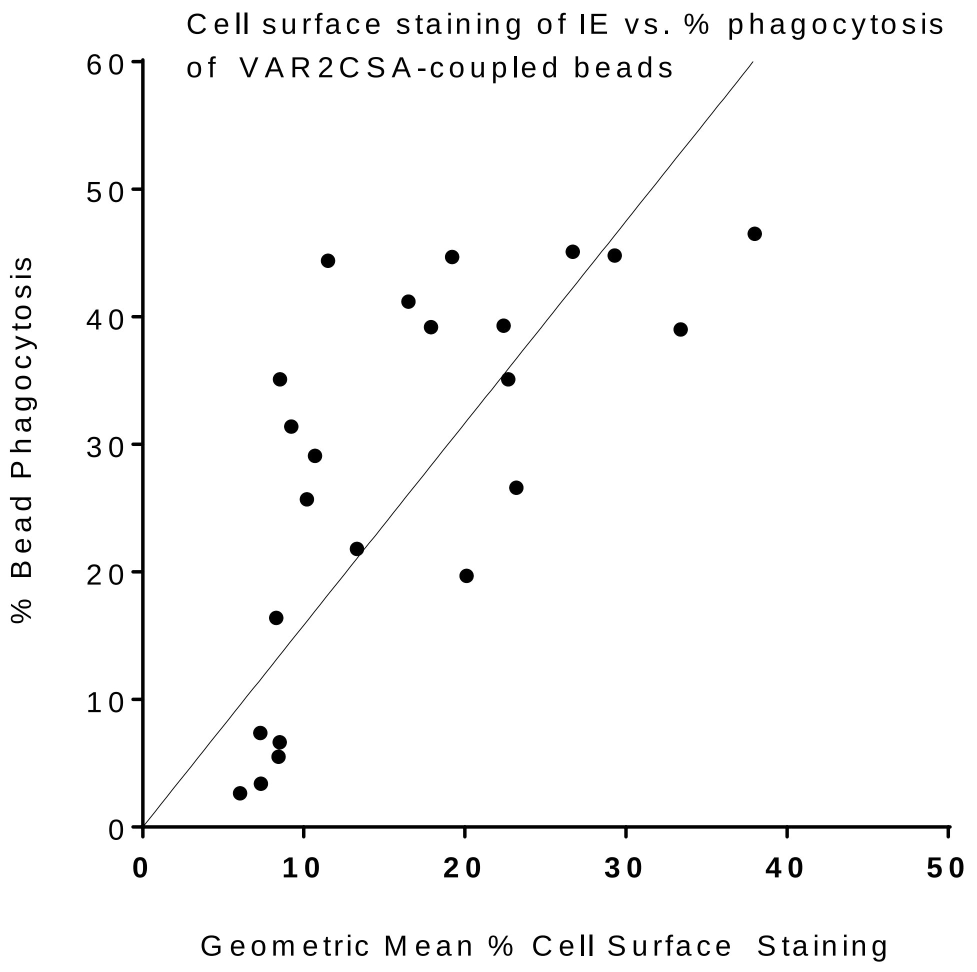

Supplemental Figure S4. Correlation between cell surface staining of IE and the geometric mean of percentage of THP-1 cells that phagocytosed VAR2CSA-coupled fluorescent beads
